# Chitosan-loaded piperlongumine nanoparticles and kaempferol enhance the anti-cancer action of doxorubicin in targeting of Ehrlich solid adenocarcinoma: in vivo and in silico modeling study

**DOI:** 10.1007/s12032-023-02282-5

**Published:** 2024-01-23

**Authors:** Fawziya A. R. Ibrahim, Neveen A. Hussein, Aisha Y. M. Soliman, Thanaa I. shalaby, Mona M. Rashad, Noura A. Matar, Tarek S. El-Sewedy

**Affiliations:** 1 Department of Applied Medical Chemistry, Medical Research Institute, Alexandria University, 165 El-Horreya Avenue, El-Hadara, Alexandria, Egypt; 2 Faculty of Applied Medical Sciences, Pharos University, Alexandria, Egypt; 3 Department of Medical Biophysics, Medical Research Institute, Alexandria University, Alexandria, Egypt; 4 Department of Histochemistry and Cell Biology Medical Research Institute, Alexandria University, Alexandria, Egypt

**Keywords:** Piperlongumine, Kaempferol, Doxorubicin, Chitosan nanoparticles, In silico

## Abstract

Doxorubicin is a chemotherapeutic drug that generates free radical-induced toxicities. Natural agents are used to potentiate or ameliorate the toxicity of chemotherapy. None of the studies investigating whether antioxidants or prooxidants should be used with chemotherapy have addressed their efficacy in the same study. Therefore, the aim of this study was to investigate the potential synergy between doxorubicin and two natural rarely in vivo studied anticancer agents; the antioxidant “Kaempferol” and prooxidant “Piperlongumine” in Ehrlich tumor mice model. 77 albino mice were divided into 11 groups; Ehrlich ascites carcinoma cells were injected intramuscularly to develop solid tumors. After 14 days, intratumoral injections of single or combinations of free or Chitosan nanoparticles loaded with doxorubicin, Piperlongumine, and Kaempferol were performed. Tumor Characterization of nanoparticles was measured, tumors were histopathologically examined and evaluation of expression for cancer-related genes by real-time PCR. In silico molecular docking was performed to uncover potential novel targets for Piperlongumine and Kaempferol. Despite receiving half of the overall dose compared to the free drugs, the combined doxorubicin/ piperlongumine-chitosan nanoparticles treatment was the most efficient in reducing tumor volume; down-regulating Cyclin D1*,* and BCL2; as well as the Beclin-1, and Cyclophilin A genes modulating growth, apoptosis, autophagy, and metastasis, respectively; up-regulating the Glutathione peroxidase expression as a defense mechanism protecting from oxidative damage. When combined with doxorubicin, Kaempferol and Piperlongumine were effective against Ehrlich solid tumors. However, the combination with the Piperlongumine-loaded chitosan nanoparticles significantly enhanced its anticancer effect compared to the Kaempferol or the same free compounds.

## Introduction

Doxorubicin (DOX) is an antibiotic that is widely used to treat solid tumors such as breast, lung, and thyroid cancers. DOX is a powerful reactive oxygen species (ROS) generator, its primary mechanism of action involves ROS-mediated oxidative damage to DNA, which results in toxicity and cellular death [1]. Although DOX is one of the most efficient chemotherapeutic agents, its therapeutic is relatively hindered by the development of ROS-induced toxicities, particularly during long-term therapy. Nephrotoxicity and hepatotoxicity are among the most common side effects observed during long-term treatment with DOX [2].

Combination therapy is rationalized by the idea that using two or more drugs with various modes of action may enhance the effectiveness of the chemo drug, and/or reduce its intolerable side effects by reducing its effective therapeutic dose [3].

Piperlongumine (PL) is a natural alkaloid isolated from Piper longum Linn fruit with a wide range of therapeutic potential [4]. The PL’s potential as an anticancer agent was reported in vitro against various solid tumor cell lines. However, in vivo studies are barely reported Notably, ROS induction by PL is a major mechanism of action for its anticancer effects [5].

Kaempferol (KMP) is a tetrahydroxyflavone that is found in various herbal seeds, leaves, flowers, and vegetables. Kaempferol’s therapeutic and preventive effects against different diseases including cancer were previously reported [6]. Similar to PL, the anticancer effect of KMP was widely reported in vitro but is rarely examined in vivo. The antioxidant and ROS scavenging power of KMP was described as a mechanism of action for its anticancer effect [7]

Anticancer therapy using pro-oxidants or dietary antioxidants has been a source of controversy, debating whether antioxidants should be used in combination with chemotherapy or not at all [8–11]. This is because as mentioned earlier, reports showing prooxidants like PL and antioxidants like KMP act as anticancer agents by modulating the cellular redox homeostasis in seemingly opposite ways.

Chitosan (CS) is an environmentally friendly biological macromolecule with a wide range of anticancer properties, and chitosan nanoparticles “CSNPs” were previously reported in treating various types of cancer [12].

As mentioned above, the anti-cancer effects of KMP and PL has barely been reported in vivo and has never been investigated in combination with DOX in vivo. Therefore, an intramuscularly implanted Ehrlich ascites carcinoma (EAC)-bearing mice model was selected in the current study to investigate the therapeutic effect, toxicity, and the implicated potential mechanisms of cell death when combining DOX with KMP or PL, either free or loaded on chitosan nanoparticles (CSNPs). Moreover, we have investigated the simulated binding of KMP and PL against the best-fitted molecular targets using in silico molecular docking analysis to identify potentially novel mechanisms of action for both molecules.

## Materials and methods

### Chemicals and reagents

Low molecular weight CS with a degree of deacetylation ≥ 85% (CAS No. 9012-76-4) and sodium tripolyphosphate (TPP) (CAS No. 7758-29-4), DXN (CAS No. 25316-40-9) were purchased from Sigma-Aldrich, Saint Louis, MI, USA. Both PL (CAS No. 20069-09-4) and Kmp (CAS No. 520-18-3) were purchased from Target Mol Chemicals Inc., Wellesley Hills, MA, USA.

### Preparation of nanoparticles

NPs were prepared as was described by Hagras et al., using ionotropic gelation utilizing the interaction between the positively charged amino groups of CS and the negative groups of TPP [13].

For the preparation of CSNPs, CS solution (1 mg/mL) was prepared by dissolving 40 mg of CS in 40 mL of acetic acid 1% (v/v), and stirred at 300 rpm, overnight at room temperature with mild stirring (300 rpm at room temperature). The pH of the solution was adjusted to 5.5 using 1 M NaOH aqueous solution with continuous stirring for 30 min. Finally, the supernatant was completely removed from the collected sediment harboring the CSNPs.

The DXN-CSNPs and PL–CSNPs were prepared by adding 3.5 mL of DXN solution (2 mg/mL) and PL solution (3.0 mg/mL anhydrous ethanol), respectively, to CS solution (1 mg/mL acetic acid, 1%), and stirring at 1000 rpm and stirred for 1 h at 300 rpm. Finally, for optimal ionic gelation, All NPs suspensions were maintained at ambient temperature overnight, then centrifuged at 14,000 rpm for 20 min to obtain the NP-containing sediment.

### Characterization of nanoparticles

The shape and size of NPs were investigated using the transmission electron microscope (TEM, JEOL X100, Japan). The potential interaction between the constituents within the NPs system was analyzed by Fourier transform infrared (FT-IR) spectroscopy (Shimadzu 8400S). FT-IR spectra were recorded on a Fourier Transform Infrared spectrophotometer using the KBr method in a spectral range of 400–4000 cm^−1^ [14]. The entrapment efficiency (EE) is expressed as a ratio between the concentration of drug-loaded on CS and the overall drug concentration. To assess the EE for DXN in NPs, the free DXN in the supernatant was measured using a UV spectrophotometer at 481 nm. To measure the EE of PL, free PL was measured using a UV spectrophotometer at 328 nm. The EE was calculated by the equation: EE = “Ct-Cu/Ct × 100”, where Ct is the overall drug concentration and Cu is the supernatant-free drug concentration.

### Animals

All animal handling procedures and operations were carried out following the International Guiding Principles for Biomedical Research Involving Animals [15]. The study was authorized by the Medical Research Institute, Alexandria University’s Institutional Animal Care and Use Committee (Approval Number: AU-122112411). Seventy-seven Swiss albino adult male mice weighing approximately 20–30 g and 2 weeks aged were acquired via the animal facility of the Medical Technology Center, MRI, Alexandria University.

### Ehrlich solid tumor (EST) induction

Balb/C female mice harboring the Ehrlich ascites carcinoma (EAC) cells were purchased from Cairo University’s Egyptian National Cancer Institute. These mice served as the major source of EAC cells to be used in solid tumor induction. Two mL of the ascitic fluid was collected from the EAC carriers’ abdominal cavity, and the EAC cells were collected by centrifugation at 400×*g*, diluted with PBS, and counted using a Neubauer hemocytometer and the trypan blue dye exclusion method. The final volume of cells was subsequently adjusted to subcutaneous injection of each mouse with a single dose of 2.5 × 10^6^ cells in 0.2 mL PBS for solid tumor induction.

### Experimental design and treatment

After the EST induction (14 days) that was confirmed by histopathological examination, the therapy started when the larger diameter of the tumor reached 1 cm in length were observed in all EAC-implanted mice. In total, the 77 mice were randomly divided into 11 groups (7 mice/group) as follows; a normal healthy group serving as negative control, an EST-bearing group receiving no treatment serving as positive control and 9 treated groups receiving: CSNPs (20 mg/kg), DXN (8 mg/kg), DXN-CSNPs (8 mg/kg), PL (2.5 mg/kg), PL-CSNPs (2.5 mg/kg), PL (2.5 mg/kg) + DXN (8 mg/kg), PL-CSNPs (2.5 mg/kg) + DXN-CSNPs (8 mg/kg), Kmp (12 mg/kg), Kmp (12 mg/kg) + DXN (8 mg/kg). All mice groups received the corresponding treatment in 0.2 ml intratumoral (IT) injections that were carried out daily for all groups containing the free drugs and every 48 h for groups containing CSNPs for a total period of 10 days. The used doses were determined based on in vivo preliminary and published reports [16–19], and were non-toxic as the survival rate for mice was 100% by the end of the experiment.

### Measurement of tumor volume

Tumor length and width were recorded using a Vernier caliper at three intervals: Zero (at the end of the 14-day tumor induction period and before treatment), and at days 5 and 10 of treatment. Measurements were performed at the same fixed time every day throughout the experiment. The volume of the tumor was calculated by measuring the diameters along the two biggest dimensions using the standard equation: *V* = *a* × (*b*)^2^/2, where *V* represents the volume in mm^3^, *a* is the larger diameter (mm), and *b* is the smaller diameter (mm) [20].

### Blood and solid tumor sampling

Twenty-four hours after the completion of the treatment period, mice were euthanized by decapitation under sodium Isoflurane (1%) anesthesia and blood samples were obtained by cardiac puncture. Blood was allowed to clot at room temperature for 20 min and sera were separated by centrifugation at 4000 rpm for 20 min at 4 °C, and stored at − 80 °C till used for biochemical analysis.

Additionally, samples from the EST were excised and divided into two portions; the first was preserved in 10% formalin, processed, and then embedded in paraffin and sections were stained by hematoxylin and eosin (H&E) staining for histopathological examination, and the second was stored at − 80 °C and used for gene expression analysis.

### Serum biochemical parameters

Liver aspartate transaminase (AST) and alanine transaminase (ALT), and kidney function parameters including urea, and creatinine levels were measured using commercial kits purchased from Biodiagnostic Co. (Giza, Egypt). The serum Total oxidant status (TOS) was measured using a commercial colorimetric kit (Elabscience Biotechnology, Texas, USA). All procedures were performed according to the manufacturer’s instructions.

### Evaluation of gene expression

The relative expression of cyclin D1 (*CCND1*), B-cell lymphoma 2 (*Bcl2*), cyclophilin A (*CyPA*), beclin-1 (*BECN1*), and glutathione peroxidase 1 (*GPX1*) genes was measured using real-time PCR (RT-PCR). Total RNA was extracted from 30 mg tissue by the GENEzol™ TriRNA Pure Kit (Geneaid Biotech, Taiwan), following the manufacturer’s instructions. The extracted RNA was converted to cDNA using the Revert Aid TM cDNA Synthesis Kit (Thermo Fischer Scientific, USA). PCR reactions were carried out using 2 μl of cDNA, 1.25 μl of each forward/reverse primer (10 pMol/µl), 10 μl (TOPreal TM qPCR 2X PreMIX) SYBER Green master mix, and nuclease-free water to 20 μl total volume/reaction. The amplification program was as follows; initial denaturation for 15 min at 95 °C, followed by 35 repeated cycles of denaturation for 10 s at 95 °C, annealing for 15 s at 60 °C, and 30 s for final elongation at 72 °C. Primer sequences used in the RT-PCR were; *CCND1* (forward) 5′ CTCCGTATCTTACTTCAAGTGCG 3′, *CCND1* (reverse) 5′ CTTCTCGGCAGTCAAGGGAA 3′; *Bcl2* (forward) 5′ GGAGAAAGGCGCTTTGG 3′, *Bcl2* (reverse) 5′ TGAGTCTCCCCTCTTCTCGG 3′; *CyPA* (forward) 5′ GCTTTTCGCCGCTTGCT 3′, *CyPA* (reverse) 5′ CTCGTCATCGGCCGTGAT 3′; *BECN1* (forward) 5′ CAGCCTCTGAAACTGGACACGA 3′, *BECN1* (reverse) 5′ CTCTCCTGAGTTAGCCTCTTCC 3′; *GPX1* (forward) 5′ GGGACTACACCGAGATGAACGA 3′, *GPX1* (reverse) 5′ ACCATTCACTTCGCACTTCTCA3′; *β-actin* (forward) 5′ GTGACGTTGACATCCGTAAAGA 3′, *β-actin* (reverse) 5′ GCCGGACTCATCGTACTCC 3′. Gene expression was calculated using the 2^−∆∆ct^ method after normalization to the internal *β*-actin housekeeping gene.

### Molecular docking

Virtual screening and selection of target protein sites for Kmp and PL were performed by the MOE 19.0901 software [21]. The best-fitted targets (AKT1, NIK, Pi3 kinase, SRC-Kinase, and HDAC6 CD1) were selected for further processing due to their vital role in the progression of the tumorigenesis process. The binding sites were obtained from co-crystallized ligands in crystal proteins (PDB codes: 3QKK- 5T8P-4BFR- 2BDJ- 5G0G) available on www.rcsb.org. The examined compounds’ two-dimensional structures were drawn in Chem-Bio Draw Ultra17.0, processed by MOE 19.0901 software, and protonated to form 3D structures [22]. Minimized structures were built for docking utilizing the prepared ligand protocol, the best-fitted postures’ docking scores with the active site were captured, and a 3D view was generated using the Discovery Studio 2019 Client program.

### Statistical analysis

The IBM SPSS software version 20.0 (Armonk, NY: IBM Corp) was used to analyze the data statistically. The Shapiro–Wilk test was used to ensure that the data was normally distributed. Mean, standard deviation, and standard error were used to describe quantitative data. The statistical significance of the results was determined at the 5% level. For normally distributed quantitative variables, the *F*-test (ANOVA) was utilized for comparing more than two groups. To assess tumor size across multiple periods, ANOVA with repeated measurements was employed. For abnormally distributed quantitative variables comparing two investigated groups, the Kruskal Wallis test was applied.

## Results

### Characterization of nanoparticles

#### Transmission electron microscope (TEM)

**Fig. 1:**
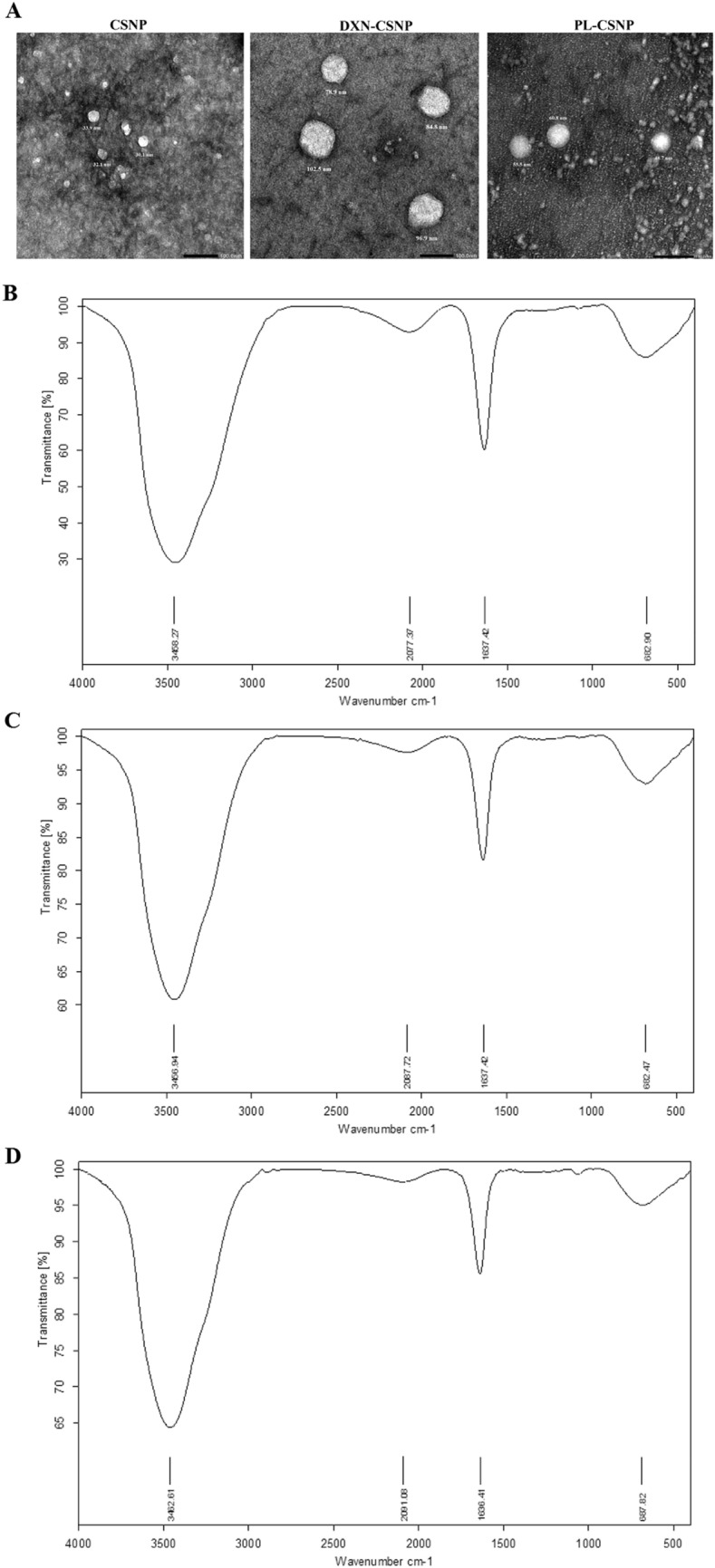
Nanoparticles characterization: **A** Representative transmission electron microscope images of the prepared chitosan nanoparticles (CSNPs), Doxo-loaded chitosan nanoparticles (DXN-CSNPs), and Piperloungumin-loaded chitosan nanoparticles PL-CSNPs, showing the shape and particle size range (nm). Analysis of the FTIR spectrum: **B** chitosan nanoparticles. **C** Doxo-loaded chitosan nanoparticles; and **D** Piperloungumin-loaded chitosan nanoparticles. (Shimadzu 8400S, FT-IR spectroscopy)

The morphological studies of NPs were carried out by TEM. The TEM images such as those shown in Fig. 1A indicate the spherical shape of the prepared NPs. From particle size analysis data, DXN and PL incorporation increased the particle size of NPs as compared to unloaded NPs. The empty NPs had a particle size range of 19–33 nm. While the DXN-CSNP ranged from 79.99 to 102.56 nm. The PL-CSNP had a size range of 43–60 nm.

#### Fourier transform infrared (FTIR) spectroscopy analysis

The CSNP, DXN-CSNP, and PL-CSNP spectra were analyzed using FTIR spectroscopy to determine their characteristic peaks corresponding to their characteristic functional groups as shown in (Fig. 1 B–D). For blank CSNP shown in (Fig. 1B), the broadband maximum was detected at 3454.27 cm^−1^ which corresponds to OH vibration representing the hydrogen bonds between the hydroxyl groups of CS and TPP. The peak for the asymmetric stretch of C–O–C was found at approximately 1100 cm^−1^, and the peak at 1387 cm^−1^ belonged to the C–N stretching vibration of type I amine. The amino band at 1637.11 cm^−1^ indicates the ionic interaction between TPP and the NH_2_ group of CS.

The FTIR spectrum for DXN-CSNP showed multiple peaks at 2087 cm^−1^ (C–H), 1637 cm^−1^ (N–H), and 1030 cm^−1^ (C–O), (Fig. 1C). These results indicate that DXN was successfully loaded onto the CSNPs. The formation of PL-CSNP by ionic gelation was tested by FTIR spectroscopy. The incorporation of PL in the CSNP was verified by a shift of the peaks at 1637 cm^−1^ found in the spectra of CSNP to 1640 cm^−1^ in the PL-CSNP spectra, **(**Fig. 1D**)**.

#### Entrapment efficiency (EE)

The absorbance intensity of unloaded DXN in the supernatant was 2.49 at *λ*_max_ of 484 nm. The corresponding concentration to the measured absorbance was 0.8 mg/ml, as obtained from the standard curve and the LE was 88.5%. On the other hand, the absorbance intensity of the unloaded PL in the supernatant was 3.0 at *λ*_max_ of 340 nm. The corresponding concentration to this absorbance was 0.58 mg/ml, calculated from a standard curve, and the EE was 80.6%, calculated as previously mentioned in the materials and methods.

### Evaluation of antitumor activities of NPs

**Fig. 2:**
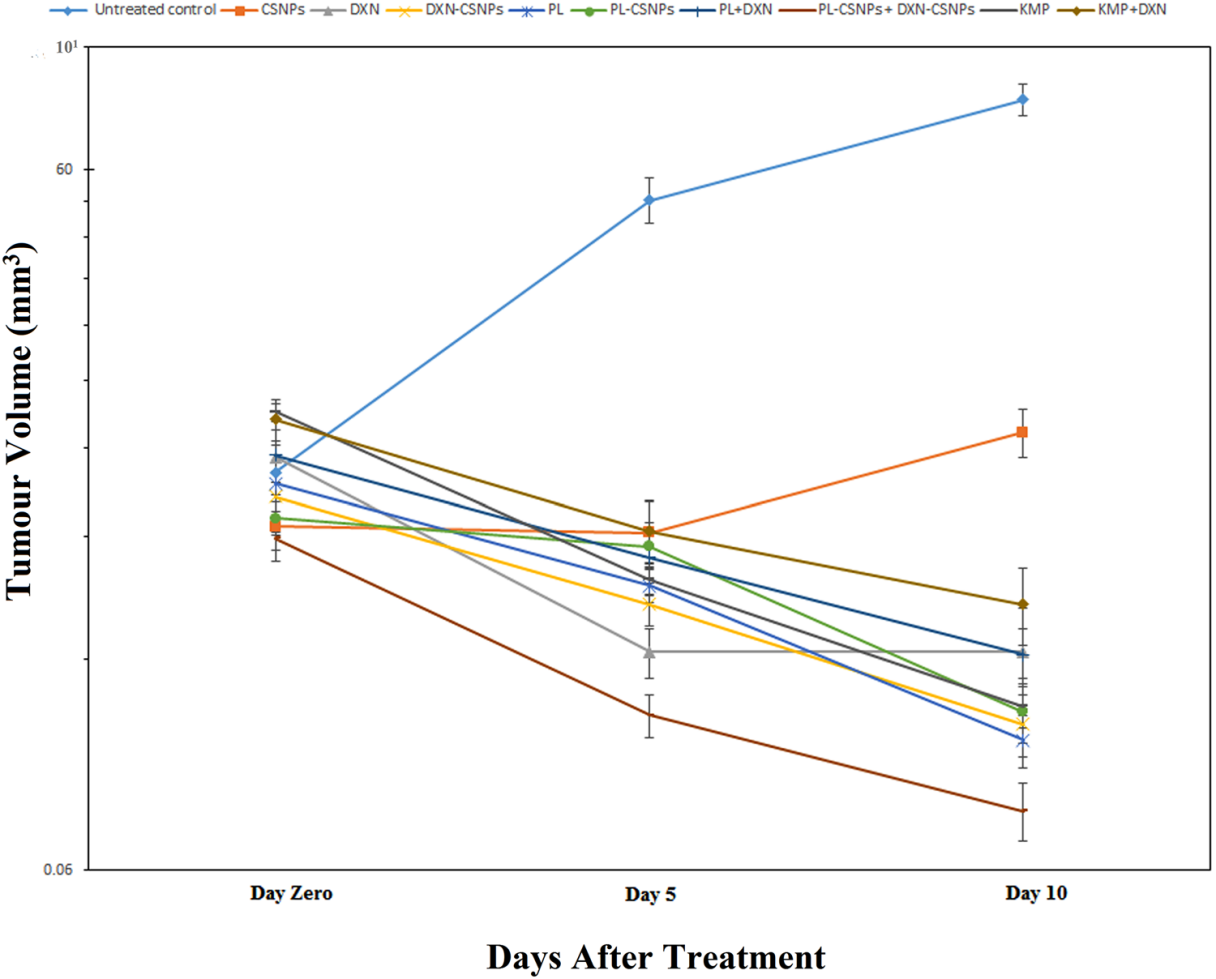
Regression of EAC subcutaneous tumor volume after treatment: Graph showing the time course of the EST tumor volume (logarithmic scale) in treated and untreated mice groups throughout a 10-day treatment period, represented as mean ± SEM (*n* = 7 mice/group)

The effect of the treatment was evaluated in vivo by monitoring the alteration in EST volume. Compared to the EST-untreated control group, all therapies had resulted in a significant (*p* ≤ 0.05) reduction in tumor volume by the conclusion of the 10-day treatment period (up to tenfold tumor reduction). Despite receiving half of the overall dose compared to mice groups treated with free DOX or PL, mice treated with the combination PL-CSNP + DXN-CSNP showed the highest reduction in tumor volume compared to EST-untreated control (0.100 ± 0.014 and 0.543 ± 0.038), and (0.073 ± 0.005 and 0.759 ± 0.065), 5 and 10 days post-treatment, respectively, (Fig. 2).

### Effect of treatment on gene expression

**Fig. 3:**
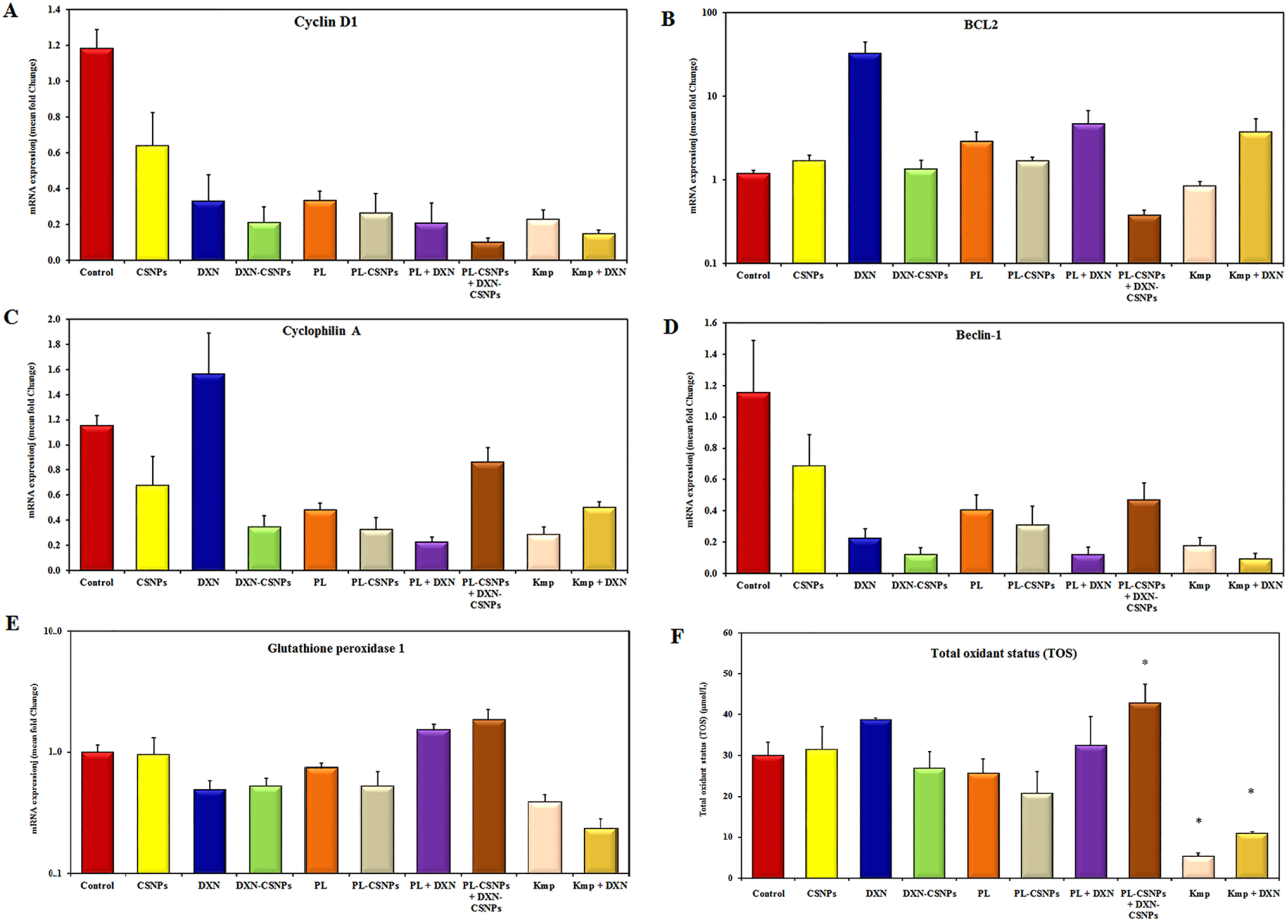
Effect of treatment on the gene expression. Relative gene expression for **A** cyclin D1, **B** Bcl2, **C** cyclophilin A, **D** Beclin-1, and **E** GPX1 genes was measured by qRT-PCR. The expression level was calculated relative to the housekeeping gene* β*-actin gene. Data are expressed as mean ± SEM (*n* = 7 mice/group). **F** Serum total oxidant status (TOS) levels in treated and control groups. Data are expressed as mean ± SEM (*n* = 7 mice/group). * means significantly different compared to the control group (*p* ≤ 0.05)

The effect of the different treatments on the expression of genes regulating key processes associated with tumorigenesis was investigated by qRT-PCR, (Fig. 3). Except for the CSNP-treated group, the expression of the tumor-promoting *CCND1* gene was significantly (*p* ≤ 0.05) downregulated in all treated groups compared to the EST-untreated control group, The PL-CSNP + DXN-CSNP combination showed the highest downregulation (tenfold) (Fig. 3A). The only significant (*p* ≤ 0.05) reduction in expression of the anti-apoptotic *Bcl2* gene was caused by the combination PL-CSNP + DXN-CSNP (45%). However, all the other treatments upregulated the *Bcl2* gene expression compared to the control untreated EST-tumor group (Fig. 3B). The pro-tumorigenic, key determinant *CyPA* gene was significantly downregulated in all treated groups compared to the untreated EST-tumor group, the most downregulation was detected in PL + DXN (80%) (Fig. 3C). Finally, the pro-autophagic *BECN1* gene expression was significantly (*p* ≤ 0.05) downregulated in DXN-CSNP, PL-CSNP, PL + DXN, Kmp, and Kmp + DXN groups as compared to the untreated EST-tumor group (Fig. 3D).

Collectively, the qRT-PCR results revealed that the various treatments modulated cell growth, survival, proliferation, and metastasis-enhancing genes to different extent depending on the combination being used, showing PL-CSNP + DXN-CSNP as the most efficient in suppressing cell proliferation and enhancing apoptosis.

### Assessment of GPX1 expression and total oxidant status

To assess the effect of treatment on the oxidative stress status and one of its major modulating enzymes, the TOS level was determined by a colorimetric assay, and the expression level for the *GPX1* gene was investigated by qRT-PCR. Compared to the untreated EST-tumor group, the *GPX1* gene expression was significantly downregulated in Kmp + DXN, and Kmp (*p* ≤ 0.05). On the other hand, combined PL-CSNP + DXN-CSNP treatment significantly upregulated the *GPX1* expression compared to all other treatments except for PL + DXN treatment (Fig. 3E). Concordant with the GPX1 gene expression, the TOS level significantly declined in both the Kmp alone and Kmp + DXN groups as compared to the control and all other treatment groups (Fig. 3F) and significantly increased in PL-CSNP + DXN-CSNP and PL + DXN treatments.

As a whole, most treatments significantly reduced both the TOS level and *GPX1* gene expression, with PL-CSNP + DXN-CSNP being the most effective in elevating both the expression of *GPX1* and the TOS level.

### Molecular docking

Based on the previous results showing promising therapeutic effects, molecular docking studies were carried out to screen for probable tumor-promoting targets that might be inhibited by binding of Kmp or PL into their active site with high affinity and stability, therefore identifying potential novel modes of action for both compounds. Table 1 depicts the binding affinity expressed as the binding energy (kcal/mol), stability as measured by the root mean square deviation (RMSD), and bond interactions between Kmp or PL and their best-fitted potential targets.

**Table 1:** Molecular docking analysis showing root mean square deviation (RMSD), docking score (kcal/mol), and interactions for Kaempferol or Piperlongumine against potential targets

Targets	Tested compounds	RMSD value (Å)	Binding energy (kcal/mol)	Interactions	
				H.B	Pi -interaction
AKT1	Kaempferol	0.80	− 6.81	2	2
	Co-ligand (3QKK)	0.59	− 6.90	3	6
*NIK*	Kaempferol	0.55	− 6.29	1	6
	Piperlongumine	0.96	− 7.06	3	3
	Co-ligand (5T8P)	0.21	− 6.50	2	11
PI3K_β_	Kaempferol	1.13	− 6.28	3	6
	Co-ligand (4BFR)	0.90	− 6.45	3	11
SRC-kinase	Kaempferol	0.90	− 6.74	3	2
	Piperlongumine	1.22	− 7.37	3	3
	Co-ligand (2BDJ)	0.65	− 7.65	4	11
HDAC6 CD1	Kaempferol	1.36	− 5.56	2	5
	Co-ligand (5G0G)	1.12	− 5.92	3	4

#### Docking of Kmp into AKT1 active site

**Fig. 4:**
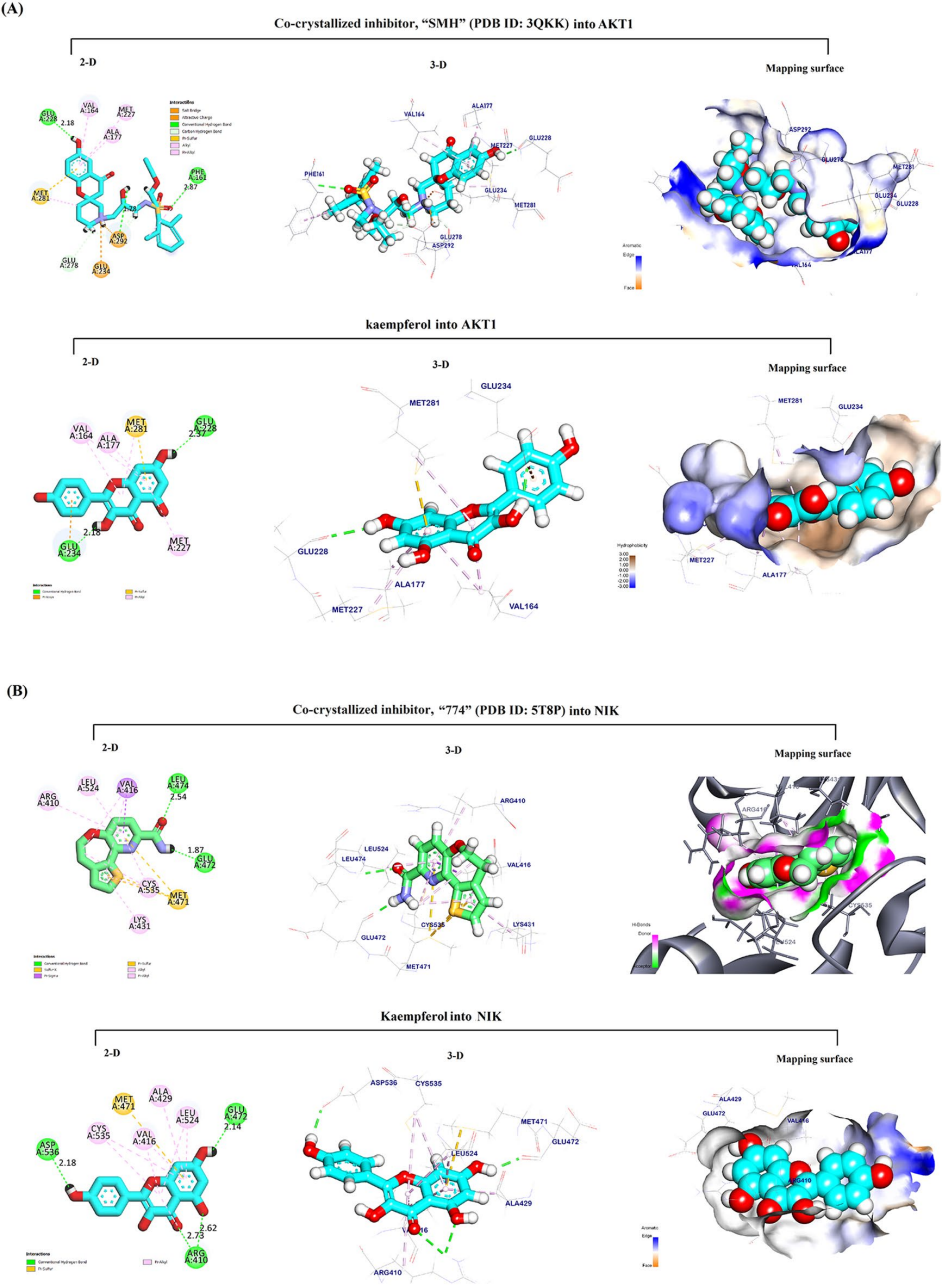

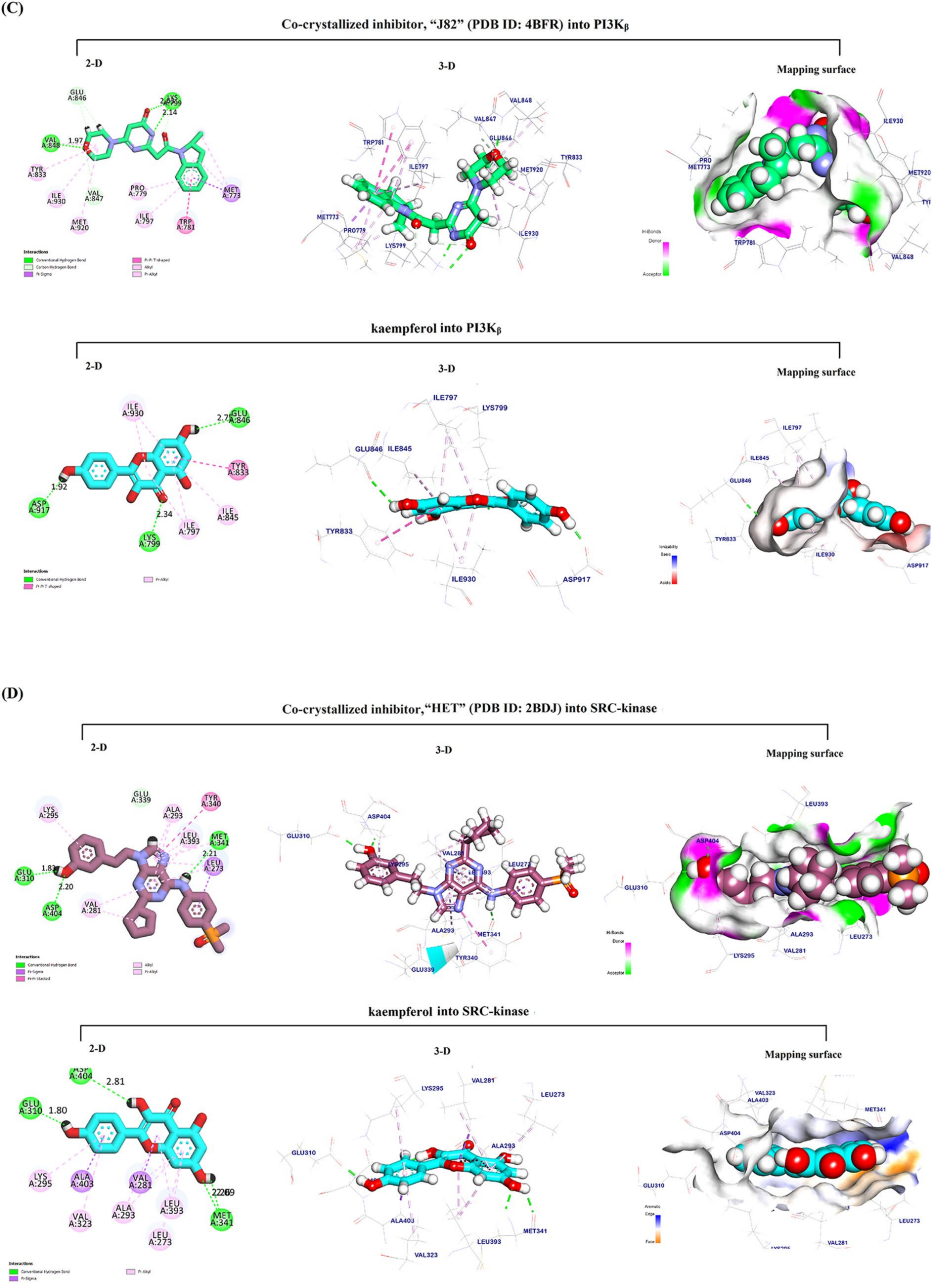

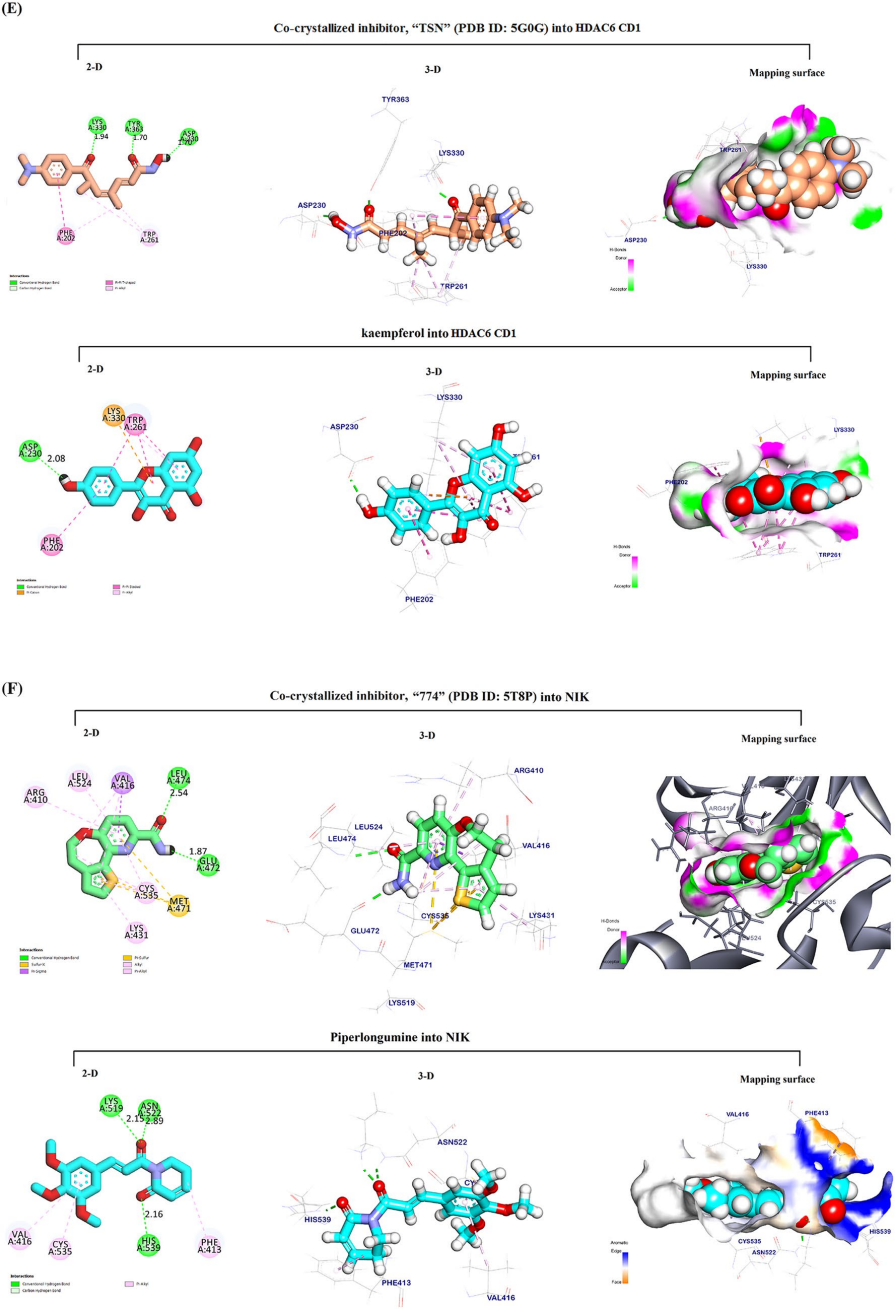

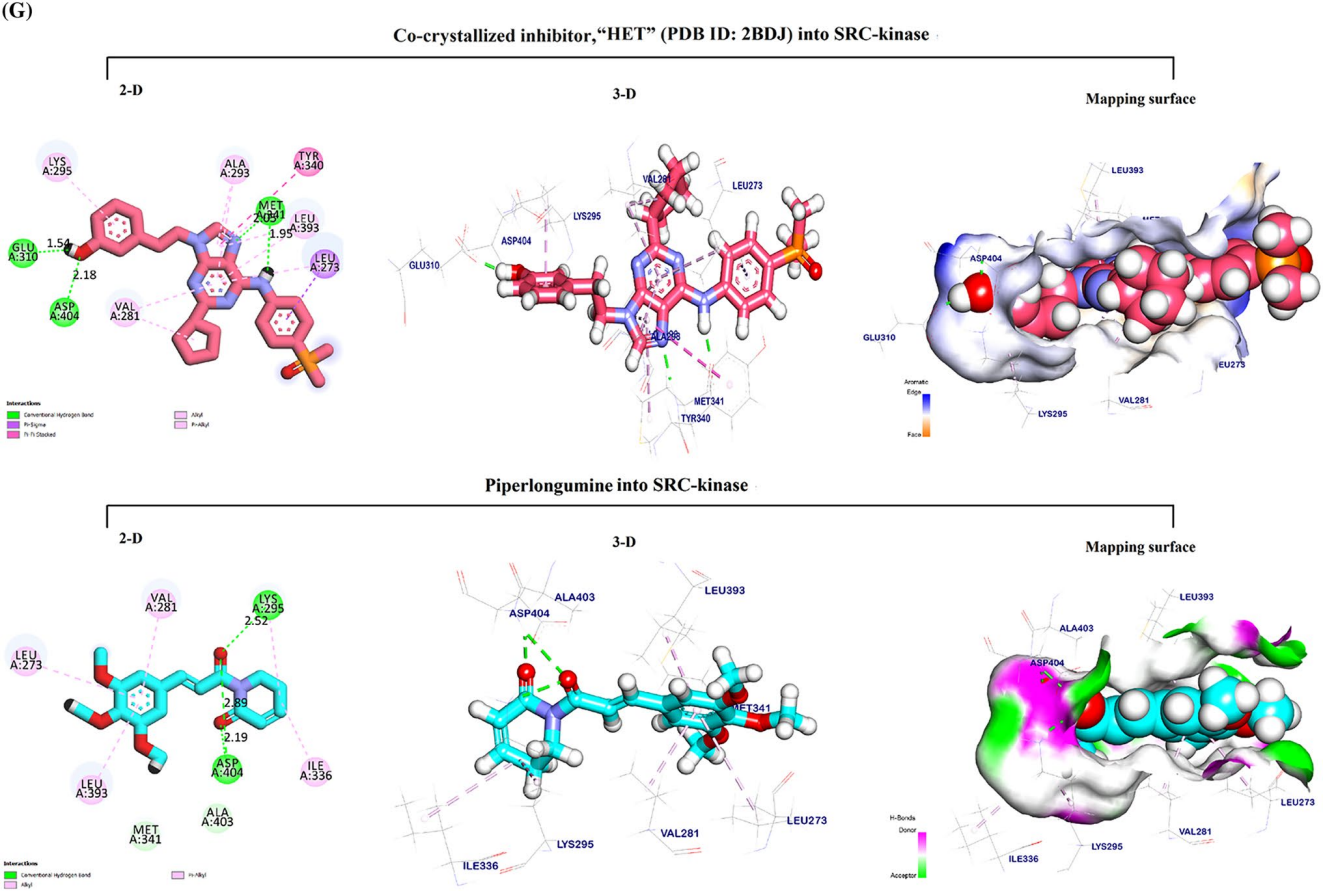
In silico Molecular docking analysis. Visualization (2-D, 3-D, and mapping surface) of the best-fitted binding poses for: Kaempferol to the active site of its potential targets **A** AKT1; **B** NIK; **C** PI3K_β_; **D** SRC-kinase; and **E** HDAC6 CD1, compared to the corresponding co-crystallized inhibitors; Piperlongumine to the active site of its potential targets **F** NIK; and **G** SRC-kinase, compared to the corresponding co-crystallized inhibitors

The docking pose for Kmp into the AKT1 active site overlapped with that of the co-crystallized inhibitor, “SMH” (PDB ID: 3QKK). This co-crystalized inhibitor had an affinity-binding energy of − 6.90. The interaction included three hydrogen bonds, six Pi-sulfur and Pi-Alkyl interactions, and two ionic interactions (Fig. 4A). The binding mode of Kmp into the AKT1 active site exhibited an energy binding of − 6.81 kcal/ mol against the AKT target site with seven Pi-Alkyl, Pi-anion, and Pi-sulfur interactions, and two hydrogen bonds.

#### Docking of Kmp into the NF-κB-inducing kinase (NIK) active site

The docking pose for Kmp into the NIK active site perfectly overlapped that of the co-crystallized inhibitor, “774” (PDB ID: 5T8P). The co-crystalized ligand bound with NIK formed eleven Pi-Alkyl, Pi-sigma, Pi-Pi interactions, and two hydrogen bonds. (Fig. 4B). The binding mode of Kmp into the NIK active site exhibited an energy binding of − 6.50 kcal/mol against the NIK target site with nine Pi-Alkyl and Pi-sulfur interactions, and four hydrogen bonds.

#### *Docking of Kmp into PI3K*_*β*_* active site*

The docking pose for Kmp into the PI3K_β_ active site overlapped with that of the co-crystallized inhibitor, “J82” (PDB ID: 4BFR). The co-crystalized ligand formed eleven Pi-Alkyl, Pi-sigma, and Pi-Pi interactions, and three hydrogen bonds (Fig. 4C**)**. The binding of Kmp into the PI3K_β_ active site formed six Pi-Alkyl and Pi-Pi interactions and three hydrogen bonds.

#### Docking of Kmp into SRC-kinase active site

The docking pose for Kmp into the SRC-kinase active site matched that of the co-crystallized inhibitor, “HET” (PDB ID: 2BDJ). The co-crystalized ligand formed eleven Pi-Pi, Pi-sigma, and Pi-Alkyl interactions and four hydrogen bonds. (Fig. 4D). The binding mode of Kmp into the SRC-kinase active site exhibited an affinity binding energy of − 6.74 kcal/ mol against the SRC-Kinase target site with ten Pi-sigma and Pi-Alkyl interactions, four hydrogen bonds.

#### Docking of Kmp into HDAC6 CD1 active site

The docking pose for Kmp into Histone Deacetylase 6 catalytic domain 1 (HDAC6 CD1) active site matched that of the co-crystallized inhibitor, “TSN” (PDB ID: 5G0G). The co-crystalized ligand formed four Pi-Alkyl interactions and three hydrogen bonds. (Fig. 4E). The binding mode of Kmp exhibited an energy binding of − 5.90 kcal/ mol against the HDAC6 CD1 target site with six Pi-Alkyl, Pi-Pi, and Cation-Pi, and one hydrogen bond.

#### Docking of PL into NIK active site

The docking pose for PL into the NIK active site was similar to that described above for the Kmp, with the same co-crystallized inhibitor “774” (PDB ID: 5T8P) interactions. The binding mode of PL into the NIK active site exhibited an energy binding of − 7.06 kcal/mol against NIK target site. PL formed three Pi-Alkyl interactions and three hydrogen bonds (Fig. 4F).

#### Docking of PL into SRC-kinase active site

The docking pose for PL into the SRC-kinase active site was also similar to what was described for the Kmp, showing the same co-crystallized inhibitor “HET” (PDB ID: 2BDJ) interactions. The binding mode of PL to the SRC-kinase active site exhibited an affinity binding energy of − 7.37 kcal/ mol against the SRC-Kinase target site. PL binds with five Pi-Alkyl interactions and three hydrogen bonds (Fig. 4G).

### Evaluation of Renal and liver function parameters

**Fig. 5:**
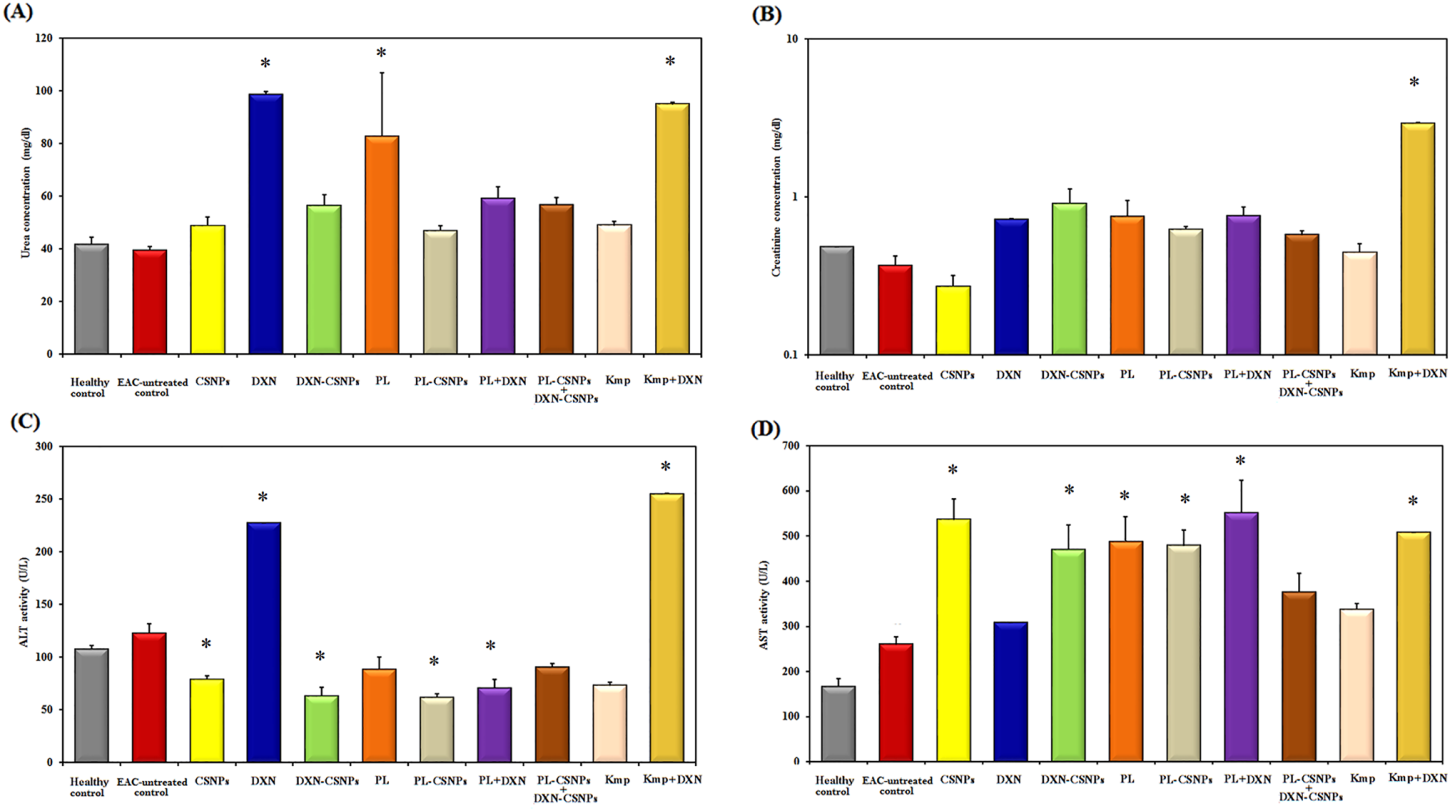
Renal and liver toxicity. Biochemical parameters of peripheral blood of Ehrlich ascites carcinoma-bearing mice were measured after a 10-day treatment period. **A** Urea concentration (mg/dL). **B** Creatinine concentration (mg/dL). **C** Alanine transaminase (ALT) activity (U/L); **D** Aspartate transaminase (AST) activity. Data are expressed as mean ± SD (*n* = 7 mice/group). * means significantly different compared to the control group (*p* ≤ 0.05)

The effect of treatment on renal and liver toxicity in healthy, untreated, and treated EST-bearing mice was assessed by measuring the serum biochemical parameters including urea and creatinine levels as markers for renal toxicity, ALT, and AST activities as liver toxicity markers**.** Among the 9 treatments performed, only the free DXN, free PL, and Kmp + DXN treatments led to a significant (*p* ≤ 0.05) increase in urea concentration as compared to the healthy or untreated EST-control groups (Fig. 5A). Except for the Kmp + DXN therapy, none of the therapies caused any significant change in creatinine levels when compared to the two control groups (Fig. 5B). A significant (*p* ≤ 0.05) decrease in ALT activity was observed after treatment with CSNP, DXN- CSNP, PL- CSNP, and PL-DXN, compared with the healthy and EST-untreated control groups (Fig. 5C). Finally, serum AST activity was mostly increased after single and combined treatments with PL + DXN causing the highest level compared to the other groups. Finally, an increase in AST activity was detected after all treatments, however, the elevation was non-significant in DXN, PL-CSNP + DXN-CSNP, and KMP treatments (Fig. 5D).

### Histopathological investigations

**Fig. 6:**
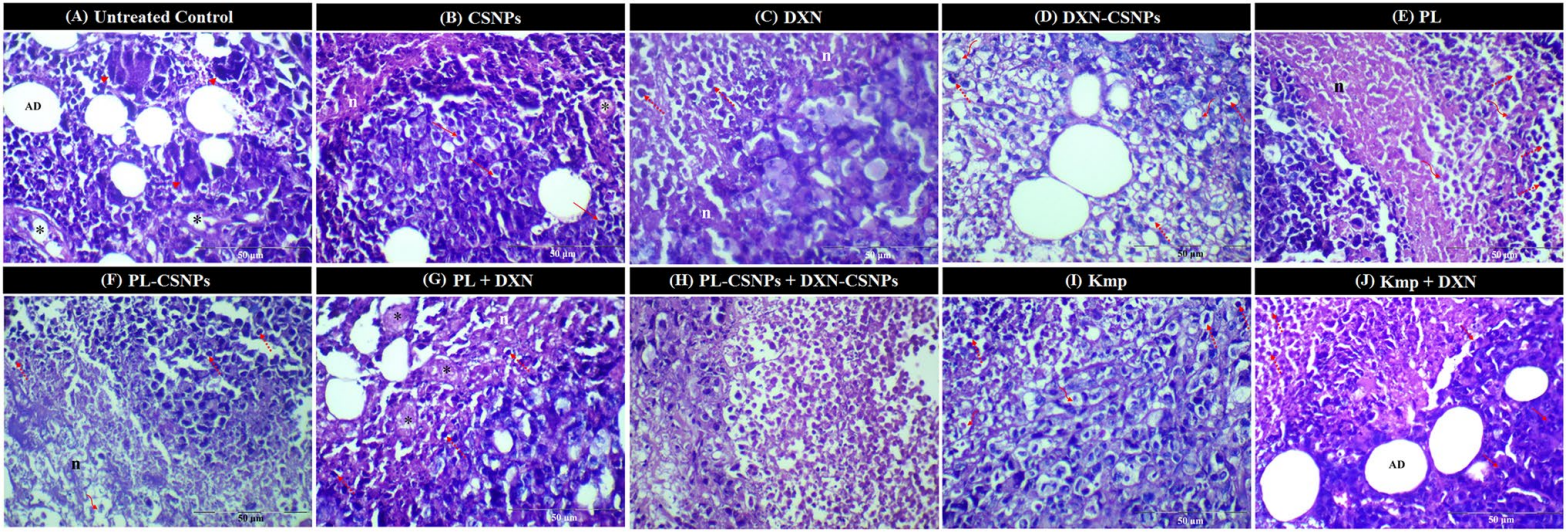
Histological study on Ehrlich Ascites Carcinoma (EAC)-bearing mice. **A** Untreated control shows extensive invasion of adipose tissue (AD) with tumor cells, newly formed large blood vessels (*), and malignant giant forms (►). **B-J** EAC-bearing mice treated with: **B** Chitosan nanoparticles (CSNPs) showing a small area of necrosis (n) surrounded by substantial tumor cells. Many mitotic figures (↑) and blood vessels (*) are noticed. **C** Doxorubicin (DXN) showing a slight decrease in the number of malignant tumor cells when compared to those treated with Kaempferol (Kmp) + DXN. A wide area of necrosis (n) and many apoptotic figures (dotted-arrow) are noticed. **D** DXN-CSNPs showing a significant reduction in the number of malignant tumor cells, expanded necrosis, a large number of apoptotic figures (dot-arrow), and few karyorrhetic nuclei (wavy arrow). **E** Piperlongumine (PL) showing a significant reduction in the number of malignant tumor cells, numerous apoptotic (dot-arrow), and karyorrhetic nuclei (wavy arrow). **F** PL-CSNPs showing a noticeable reduction in the number of malignant tumor cells, numerous apoptotic nuclei (dot-arrow), and few karyorrhetic nuclei (wavy arrow). **G** PL + DXN showing a slight decrease in malignant tumor cells compared to Kmp + DXN treatment, destructive blood vessels (*), and many apoptotic figures (dotted-arrow) are observed. **H** PL-CSNPs + DXN-CSNPs showing few scattered malignant tumor cells, and a vast number of apoptotic (dot-arrow) and karyorrhetic cells (wavy arrow). **I** KMP showing a noticeable reduction in the number of malignant tumor cells. Expanded necrosis, numerous apoptotic figures (dot-arrow), and few karyorrhetic nuclei (wavy arrow). **J** Kmp + DXN showing moderate invasion of adipose tissue (AD) with tumor cells and a wide area of necrosis. Few apoptotic (dot-arrow) and mitotic figures (↑) are observed. Sections were stained with hematoxylin and eosin (H&E), scale bar = 50 μm

CSNP treatment resulted in tiny patches of necrosis surrounded by a large number of tumor cells, as well as many mitotic figures and blood vessels. When compared to Kmp + DXN, DXN single treatment resulted in a modest decrease in the number of malignant tumor cells, large regions of necrosis and apoptotic figures were also detected. On the other hand, treatments with DXN-CSNPs, PL, and PL-CSNPs resulted in a significant decrease in the number of malignant tumor cells, a high number of apoptotic figures, damaged blood vessels. Finally, the PL-CSNP + DXN-CSNP combination resulted in a remarkable reduction in the number of malignant cells that appeared as dispersed cells concomitant with the presence of a vast number of apoptotic and karyorrhetic cells. Overall, the histopathological study supported the molecular findings and demonstrated the effectiveness of all treatments to various levels, presenting the PL-based therapies as the most efficient (Fig. 6).

## Discussion

One of the most commonly used chemotherapeutic agents for treating breast cancer and other solid tumors is DOX. The effectiveness of a combination treatment between DOX and natural agents was reported against cancer [23].

It is well-accepted that the anti-tumor effect of most chemotherapeutics drugs is due to oxidative stress and ROS-mediated cell destruction, and since ROS also activates programmed cell death [24]. Paradoxically, numerous antioxidants have been produced in recent decades and have shown therapeutic potential; nevertheless, their clinical usefulness in cancer remains uncertain and even controverted by other researchers reporting a possible pro-cancer effect for antioxidants [25, 26].

This ongoing dispute and the need for more efficient herbal-based anticancer combinations prompted our team to assess the anticancer efficacy and the possible mechanisms of action when combining DOX and two natural anticancer agents, the anti-oxidant (Kmp) or pro-oxidant (PL) used free or loaded on CSNPs against an in vivo solid mammary cancer, the EST-mice model.

The current study showed that among the different single and combination treatments performed, the greatest reduction in tumor volume was observed in EST-mice that were treated with the combination of PL-CSNPs + DXN-CSNPs **(**Fig. 2**)**. Remarkably, this effect was obtained by delivering an overall half the dose of free DOX and/or the PL to the tumor. This result is in line with strategies that are currently being developed for reducing the overall dose of chemotherapy drugs while maintaining their effectiveness to enhance the quality of life for patients. [27]. The observed enhanced anticancer effect might be more attributed to a potential synergistic effect between CSNPs, DOX, and PL rather than the CSNPs since CSNPs alone did not show the same efficiency, whether on tumor volume or cancer-modulating genes. However, CSNPs were reported to enhance the accumulation and absorption of DXN and PL [28, 29].

This DATA was the first piece of evidence suggesting that the PL loaded on the CSNPs might have the most prominent anti-proliferative effect rather than the Kmp against EST in vivo. This finding agrees with a previous study showing that treatment with PL significantly reduced tumor weight [30]. Additionally, it was reported that treatment with either PL significantly inhibited tumor growth of xenograft tumors obtained from patient-derived PNX0010 RCC cells [31].

On the molecular level, we have examined the effect of doxorubicin, Piperlongumin, and Kaempferol single and combined, free or chitosan nanoparticle-loaded therapy treatment on several genes that are known to play a central role in the pathogenesis of breast cancer including Cyclin D1 *(CCND1)*, which is a major cell cycle regulator; B-cell lymphoma 2 (*Bcl-2*), the major anti-apoptotic gene; Cyclophilin A (*CyPA*), which encodes a prolactin-binding protein that binds and therefore is considered as a key driver of breast cancer metastasis and development.; Beclin-1 (*BECN1*), is the major modulator for autophagy and tumorigenicity of breast cancer stem cells; Glutathione peroxidase 1 (*GPX1*), being reported to regulate adhesion and metastasis of breast cancer cells.

In agreement with the results mentioned above, the CSNPs loaded PL and DXN combination was the most potent in the downregulation of pro-cancer gene expression. This prominent effect was manifested by the greatest reduction in the *CCND1* and *Bcl2* gene expression after their combination treatment. PL was reported as an inhibitor of cell growth, and an inducer for cell cycle arrest by decreasing the level of Cyclins including *CCND1* [32, 33]. In 2022, the anti-cancer effective synergism between PL and DXN was shown against osteosarcoma cells via the downregulation of the *Bcl2* gene [34]. Our data concord with these results, suggesting PL-CSNPs + DXN-CSNPs as the most potent combination leading to the downregulation of the *Bcl2 gene.* Induction of cell cycle arrest which in turn leads to apoptosis was reported for PL [35] *DOX* [36], *and CSNPs* [37].

Among the genes modulating essential cancer-related processes investigated in the current study was the *CyPA gene* which is a marker protein during malignancy and is reported to play a key role in tumor development and metastasis [38]. The free PL + DXN combination and the Kmp single treatment were the most efficient in reducing the *CyPA gene expression*, To the best of our knowledge, the effect of PL or Kmp on the expression of this critical modulator gene was not explored before. Our findings provide the first evidence that *CyPA* might be one of the genes implicated in the anticancer actions of PL and Kmp.

Autophagy is a vital biological process that keeps cells in homeostasis by digesting and cycling defective or persistent proteins, misfolded proteins, or damaged and aberrant structures [39]. Autophagy plays two distinct functions in tumor development and repression in cancer. An anticancer suppressive effect during cancer initiation by eliminating defective cells. Furthermore, autophagy protects established malignant cells by fulfilling their biochemical and energy production needs throughout the advancement of cancer. Therefore, targeting autophagy in late cancer has become a promising treatment option [40].

Since subcutaneous EAC cells are highly malignant undifferentiated carcinoma, therefore, in EAC cells autophagy might support cancer cell development rather than its destruction. In this perspective, targeting and inhibition of autophagy might be considered as a goal for effective treatment. Indeed, our autophagy-enhancing *BECN1* gene expression analysis reveals that *BECN1* was significantly downregulated by most of the treatments, with PL + DXN and KMP + DXN being the most potent inhibitors. All the above-mentioned findings with the supporting studies suggest that PL might exert a potential anticancer effect by modulating apoptosis, autophagy, and cell growth and proliferation.

To further identify new potential targets for PL and Kmp that might lead to their anticancer action, we have conducted an in silico molecular docking study to gain insight into molecular binding modes, screen, and thereafter select possible target proteins for the PL and Kmp. Both PL and Kmp docked the active sites of the NF-kappaB-inducing kinase (NIK) and the Proto-Oncogene Tyrosine-Protein Kinase (SRC-kinase) successfully with a high negative energy value compared to their corresponding co-ligand inhibitors (Table 1). Considering their significant cancer-promoting role, targeting NIK [41], and SRC-kinase [42] has been recently shown as an effective anti-cancer-targeted therapy.

The common targeting of these two major signaling pathways by PL and Kmp may explain the relative similarities between PL and Kmp in some of the data presented, even though they presumably have opposing ROS effects. Interestingly, the binding energy between PL and NIK (− 7.06 kcal/mol) was higher than that of Kmp (− 6.29 kcal/mol) and the (5T8P) co-ligand inhibitor (− 6.50 kcal/mol). Similarly, the PL binding energy to SRC-kinase was higher compared to Kmp (− 7.37 and − 6.74 kcal/mol), respectively, probably explaining the relatively dominant anti-cancer effect of PL over Kmp. In addition to the above suggested common targets, Kmp docked the active sites of the AKT1 Kinase, PI3Kβ phosphatidylinositol-3-kinase, and Histone deacetylase 6 catalytic domain 1 HDAC6 CD1 with high binding energies, all of which have been identified as prospective cancer-targeting options [43–45].

Finally, our histopathology examination validated the efficacy of the PL-CSNP + DXN-CSNP combination treatment, as did the molecular data, and revealed that this combination resulted in the most significant reduction in the number of malignant cells, while greatly increasing the apoptotic cell death which was manifested by the increased number of apoptotic and karyorrhetic cells found, suggesting apoptosis might be the major cytotoxic mechanism of action.

## Conclusion

Both the prooxidant Piperlongumine and the antioxidant Kaempferol were effective against EST when combined with doxorubicin. However, the combination with the Piperlongumine-loaded chitosan nanoparticles was the most efficient in reducing tumor size and enhancing apoptotic cell death.

## Data Availability

The datasets generated during and/or analyzed during the current study are available from the corresponding author on reasonable request.

## Abbreviations

RMSD: Root mean square deviation
AKT1: Protein Kinase B
*NIK*: *NF*-*κB*-*inducing kinase*
PI3K_β_: Phosphatidylinositol-3-kinase
SRC-kinase: Proto-Oncogene Tyrosine-Protein Kinase Src
HDAC6 CD1: Histone Deacetylase 6 catalytic domain 1
